# A novel stop-gain mutation in *DPYS* gene causing Dihidropyrimidinase deficiency, a case report

**DOI:** 10.1186/s12881-020-01070-6

**Published:** 2020-06-29

**Authors:** Malihe Mirzaei, Arghavan Kavosi, Mahboobeh Sharifzadeh, Ghazale Mahjoub, Mohammad Ali Faghihi, Parham Habibzadeh, Majid Yavarian

**Affiliations:** 1 Persian BayanGene Research and Training Center, Shiraz University of Medical Sciences, Shiraz, Postal Code: 7134767617 Iran; 2 Department of Biology, Islamic Azad University, Arsanjan Branch, Arsanjan, Iran; 3 Department of Psychiatry and Behavioral Sciences, University of Miami Miller School of Medicine, Miami, USA; 4 Student Research Committee, Shiraz University of Medical Sciences, Shiraz, Iran; 5 Hematology Research Center, Shiraz University of Medical Sciences, Shiraz, Postal Code: 7134767617 Iran

**Keywords:** Dihidropyrimidnase deficiency (DHP deficiency), *DPYS*, Novel stop gain mutation, Case report

## Abstract

**Background:**

Dihidropyrimidinase (DHP) deficiency is an inherited inborn error of pyrimidine metabolism with a variable clinical presentation and even asymptomatic subjects. Dihydropyrimidinase is encoded by the *DPYS* gene, thus pathogenic mutations in this gene can cause DHP deficiency. To date, several variations in the *DPYS* gene have been reported but only 23 of them have been confirmed to be pathogenic. Therefore, the biochemical, clinical and genetic aspects of this disease are still unclear.

**Case presentation:**

Here, we report a 22-year-old woman with DHP deficiency. To identify the genetic cause of DHP deficiency in this patient, Whole Exome Sequencing (WES) was performed, which revealed a novel homozygote stop gain mutation (NM_001385: Exon 9, c.1501 A > T, p.K501X) in the *DPYS* gene. Sanger sequencing was carried out on proband and other family members in order to confirm the identified mutation. According to the homozygote genotype of the patient and heterozygote genotype of her parents, the autosomal recessive pattern of inheritance was confirmed. In addition, bioinformatics analysis of the identified variant using Mutation Taster and T-Coffee Multiple Sequence Alignment showed the pathogenicity of mutation. Moreover, mRNA expression level of *DPYS* gene in the proband’s liver biopsy showed about 6-fold reduction compared to control, which strongly suggested the pathogenicity of the identified mutation.

**Conclusions:**

This study identified a novel pathogenic stop gain mutation in *DPYS* gene in a DHP deficient patient. Our findings can improve the knowledge about the genetic basis of the disease and also provide information for accurate genetic counseling for the families at risk of these types of disorders.

## Background

Dihydropyrimidinase (DHP) deficiency is a metabolic disease which is very rare and mutations in the *DPYS* gene can be the causative reason [1]. This gene encodes an important enzyme called Dihydropyrimidinase which plays its role by breaking down the pyrimidines [2]. The pyrimidine bases (Thymine and Uracil) are broken down through a three step reductive enzymatic pathway in humans. In this process, Dihydropyrimidine Dehydrogenase (DPD), Dihydropyrimidinase and β-ureidopropionase are involved in the first, second and the third steps respectively. At first, Thymine and Uracil are converted to 5,6-dihydrothymine and 5,6-dihydrouracil, followed by the next step in which the hydrolytic ring opening of the Dihydropyrimidines are catalyzed and finally in the last step, β-aminoisobutyric acid and β-alanine are resulted from the alterations of N-Carbamyl-β-aminoisobutyric acid and N-Carbamyl-β-alanine [3–5]. In contrast to DPD, which is expressed in all tissues, DHP and β-ureidopropionase are solely expressed in kidney and liver [6].

The *DPYS* gene covers 10 exons on chromosome 8q22 which spans approximately 80 kb of the genomic DNA. The related human’s cDNA contains 1560 nucleotides open reading frames encoding polypeptides of 519 amino acids with 56.6 KDa molecular weight [7]. Mutations in this gene cause Dihydropyrimidinase deficiency (OMIM #222748) with an autosomal recessive mode of inheritance that is characterized by increased levels of dihydropyrimidine in urine (dihydropyrimidin-urea), cerebrospinal fluid and blood [8, 9]. Patients with DHP deficiency have been shown to have variable clinical presentation ranging from severe neurological malfunctions [4, 10–12] to other presenting features including dysmorphic features, failure to thrive, growth retardation and gastrointestinal problems [4, 12–17]. On the other hand, a number of asymptomatic patients with DHP deficiency have also been reported. It is still unclear why some affected people with DHP deficiency do not develop symptoms related to the condition [15–17].

This study was aim to identify a disease causing mutation in an affected Iranian woman with DHP deficiency without prior history of this condition in her family members.

## Case presentation

The proband was a 22-year-old Iranian woman (south of Iran, Fars province) with DHP deficiency whose parents are a first-degree cousins. There was not any symptoms in the proband until 20 years of age, when during her first pregnancy, elevated liver enzymes was detected during her routine laboratory evaluations (ALT of 147 U/L, reference range: < 31 U/L, AST of 108 U/L, reference range: < 31 U/L and Alk-P of 350 U/L, reference range: 64–306 U/L). She also had increased level of serum iron of 227 μg/dL, reference range: 50–170 μg/Dl and PT of 14.5 s, reference range: 10.5–11.5 s. A spontaneous abortion happened to her during the first trimester of pregnancy. She was evaluated and tested for autoimmune antibodies in which the negative results excluded autoimmune hepatitis as a disease leading to liver dysfunction. Thereafter, she was presented with bloody vomiting, diarrhea, jaundice, weight loss and bilateral mild mid frequency sensorineural hearing loss. Abdominal and pelvic ultrasonography was in favor of cirrhosis and in liver biopsy cryptogenic cirrhosis was confirmed. The patient was initially diagnosed as having glycogen storage disease by considering the clinical evaluation, and referred to genetic counseling where whole-exome sequencing was conducted to discover the underlying etiology. According to NGS results, for confirming the genotype-phenotype correlation, the patient was referred to evaluate the levels of dihydrothymine and dihydrouracil by HPLC tandem-mass spectrometry in which elevated levels of both were identified in the patient’s urine in comparison with the mean concentration observed in control group’s urine (dihydrothymine (μmol/mmol creatinine): patient = 148, controls = 1.4 ± 1.1 (*n* = 106), dihydrouracil (μmol/mmol creatinine): patient = 254, controls = 4.9 ± 3.2 (n = 106)). There was no history of such condition in other family members.

### Whole exome sequencing (WES)

All exons of protein coding regions and flanking introns belonged to the extracted DNA from the proband were captured and enriched by Whole Exome Sequencing method (Agilent sure select v5 exome capture kit). Next Generation Sequencing (NGS) method was conducted using illumina sequencing platform to sequence the libraries with mean > 80-100X coverage. The human reference genome (GRCH37/hg19) was downloaded in order to perform the alignment to Sequence reads using BWA program [18, 19]. Picard and GATK version 3.6 were used to identify the relevant variants [20, 21]. The VEP program was used to annotate the variants [22]. In order to perform further annotation a set of related disease databases including OMIM, HGMD, GWAS, SwissVar and Clinvar as well as published variants were used. Filtering of common variants was done according to allele frequency in ExAC, 1000 Genome phase3, dbSNP147 and EVS. Some bioinformatics online softwares such as SIFT, polyphen2, LRT, mutation assessor and mutation taster2 were also used to discover the disease causing variants.

### Sanger sequencing

In order to segregate the mutation, Sanger sequencing was conducted using ABI BigDye Terminator Cycle Sequencing Kit (Applied Biosystems®, USA) on extracted DNA (QIAamp DNA minikit, Qiagen, Germany) belonged to the patient and her family members (parents, sister and brother). Epoch Microplate Spectrophotometer (Bio Tek Instruments, USA) was utilized to calculate the quality of extracted DNA. Desired genomic region was amplified using following oligonucleotide PCR primer pairs: *DPYS*-E9-F: 5′-CACAAAAAGTGGGACAATCC-3′, *DPYS*-E9-R: 5′-GTGAAGCCTCTGACCTTGAT-3′. The obtained sequences were analyzed using Finch TV software and NCBI Blast.

### Quantitative real time PCR (q-RT PCR)

Liver biopsy specimens underwent RNA (total RNA) extraction via Invitrogen TRIzol Reagent (Thermo Fisher Scientific, USA) based on the company protocol. Quality of RNA was evaluated by Epoch Microplate Spectrophotometer (Bio Tek Instruments, USA). Normalized RNA samples were used to synthesis cDNA by Fermentas cDNA synthesis kit (Thermo Fisher Scientific, USA) per manufacturer’s instructions. In order to assess the *DPYS* gene expression SYBR Green Master Mix (Invitrogen) was used to perform the Real Time PCR in Rotor-Gene Q (QIAGEN, Germany) Real Time PCR cycler. The experiment was conducted in triplicate. In the current study, *GAPDH* (Glyceraldehyde 3-phosphate dehydrogenase) was used as an internal control gene. The following intron-spanning primer pairs were utilized in our study: *DPYS*-F: 5′-ACCCGACTTCCTCATGAATCT-3′, *DPYS*-R: 5′-CATCCGATCTTCAACACCATTCA-3′, for the gene of interest and *GAPDH*-F: 5′-ACAACTTTGGTATCGTGGAAGG-3′, *GAPDH*-R: 5′-GCCATCACGCCACAGTTTC-3′ for the reference gene (*GAPDH* PrimerBank ID: 378404907c2, https://pga.mgh.harvard.edu/cgi-bin/primerbank/new_search2.cgi). Comparative threshold cycle method (2^-∆∆CT^) was used to compare the relative expression of *DPYS* gene between the patient’s liver tissue and her sister’s liver tissue who genetically confirmed to be unaffected (the liver biopsy was performed in her sister due to the increased levels of ALT, AST, Alk phosphatase and she was suspected to be affected with Autoimmune hepatitis due to a positive test for ANA and ASMA (titers ≥1:160)).

## Results

NGS data revealed a novel homozygous nonsense mutation in exon nine of the *DPYS* gene that resulted in an early stop codon and premature truncation of the protein at codon 501 (*DPYS*: ENST00000351513, NM_001385: Exon9: c.1501 A > T: p.K501X). This mutation has not been reported before, so allele frequency for this mutation is not available in EXAC, 1000 Genomes, EVS and dbSNP147. There are also no reports in the literature on clinical manifestations of this mutation. Therefore, this mutation is considered as novel variant. Compound heterozygous or homozygous mutations in the *DPYS* gene have been reported in dihydropyrimidinase deficiency (OMIM #222748).

**Fig. 1:**
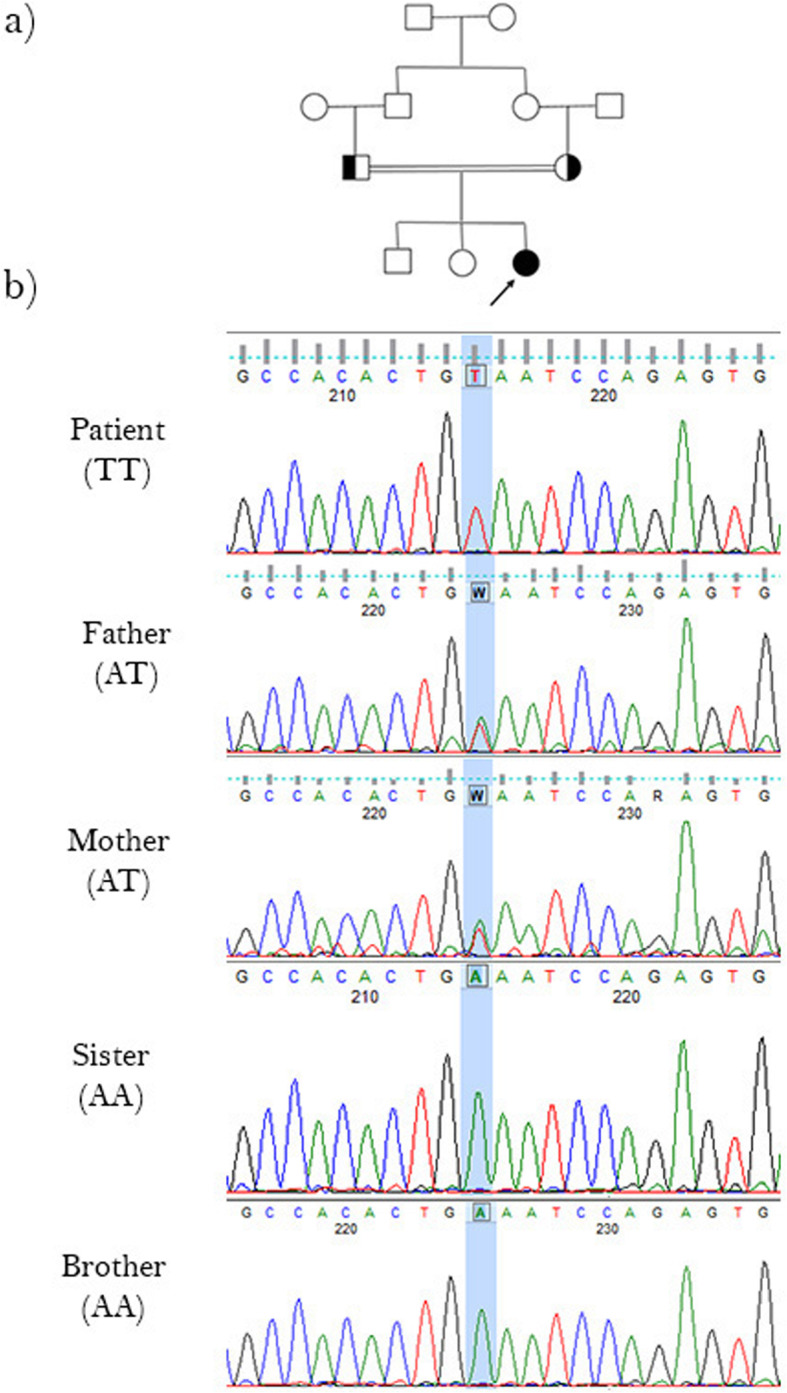
Pedigree and Sanger sequencing details of the family. **a** The proband (marked by arrow) is a 22 year-old woman who is product of consanguineous marriage. **b** The autosomal recessive pattern of inheritance was confirmed in this family using Sanger sequencing based on identified homozygote mutation in the proband and heterozygote mutation in her parents

Sanger sequencing results confirmed that the proband was homozygous for the mutant allele of this novel mutation, while her healthy brother and sister were homozygous for the wild type allele and both their mother and father were heterozygous for the mutation, which indicates the autosomal recessive inheritance pattern for this disease (Fig. 1a and b).

**Fig. 2:**
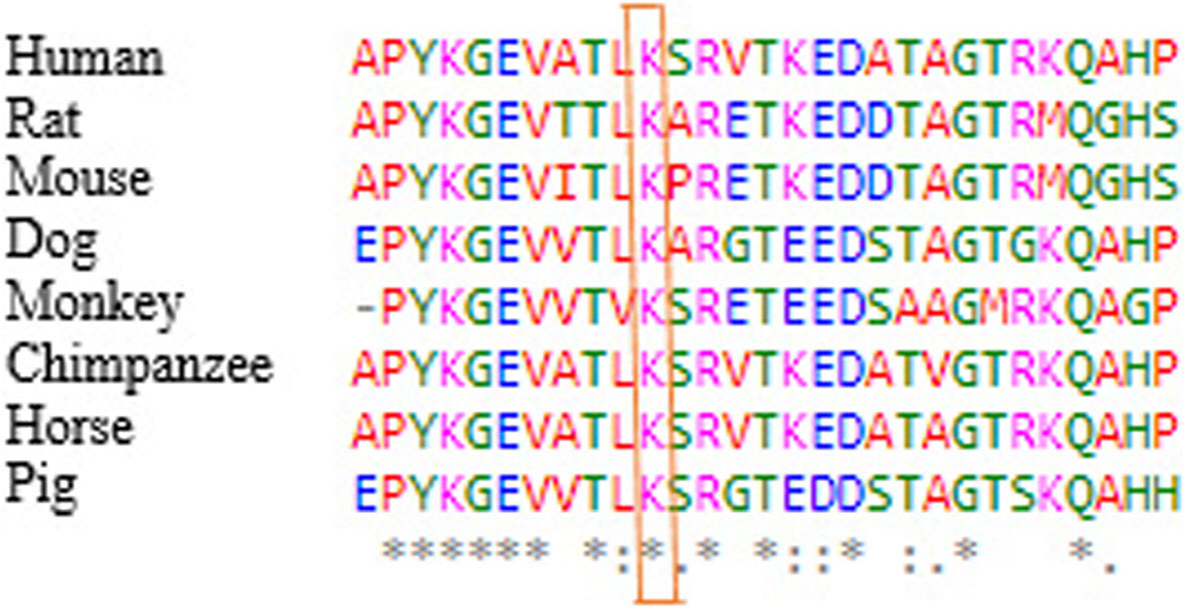
Comparative amino acids alignment of DPYS protein across mammals shows that the Lys501 residue is highly conserved during evolution. The conserved Lys (K) residue is shown in the rectangular box. National Center for Biotechnology was used to obtain protein sequences. Symbols (*) and (:) shows identical amino acids and just similar amino acids respectively

The Mutation taster tool predicts this variation as a disease causing one. The position of stop codon in wild type amino acid sequence is in codon 520, while the mutant one is in codon 501, which can lead to nonsense mediated decay and also amino acid sequence changes. The comparative sequence alignment was done across most mammals using T-Coffee Multiple Sequence Alignment Program. The results show that this amino acid is conserved in mammals during evolution and is therefore important for proper protein function (Fig. 2).

**Fig. 3:**
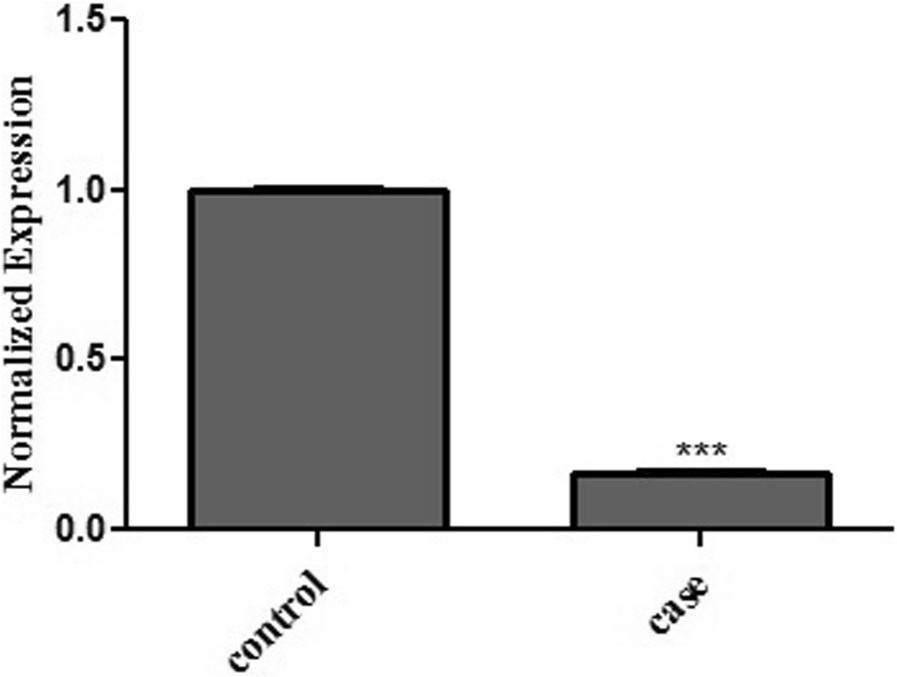
Quantitative Real-Time PCR analysis of *DPYS* expression in patients liver tissue and a healthy tissue as control. The mRNA expression level of DPYS showed a significant decrease in patients liver tissue compared to the control

Real time PCR result revealed a significant difference between the mRNA expression level of *DPYS* gene in patient’s liver tissue and the normal tissue as control. The *DPYS* gene showed about 6-fold down regulation in patients liver tissue compared to the control (Fig. 3).

## Discussion and conclusions

The *DPYS* gene encodes DHP which is the second enzyme in the catabolic pathway of pyrimidines [23]. Mutations in the *DPYS* gene can cause DHP deficiency, which is a rare inborn error of the pyrimidine degradation pathway [24]. This disorder shows variable clinical manifestations including severe neurologic involvement, dysmorphic features, feeding problems, mild intellectual disability and even asymptomatic individuals [25]. In a study investigating the clinical and molecular characteristics of 17 patients with dihydropyrimidinase deficiency, the most frequent clinical features were seizure, intellectual disability and hypotonia; gastrointestinal problems were also found to be common among affected individuals [4]. The biochemical, clinical and genetic causes of DHP deficiency remain still unclear. According to the population screening for pyrimidine metabolism disorders, asymptomatic patients with complete deficiency of DHP which is caused by mutations in the *DPYS* gene were reported. This evidence reflects the probable effects of environmental factors in causing the clinical phenotypes [25]. Although this patient was diagnosed with DHP deficiency using accurate biochemical, genetic and bioinformatics investigations, it is difficult to prove that the clinical presentations is due to the DHP deficiency.

Recent investigations show that patients with even partial deficiency of DHP are liable to developing 5-fluorouracil (5FU) toxicity [26, 27].This highlights the importance of DHP deficiency as an important pharmacogenetic disorder and calls for measuring DHP level even in asymptomatic patients before administration of 5FU.

The first patient (Turkish male infant) with DHP deficiency was reported by Duran et al. in 1991, who was diagnosed by presence of dihydrouracil and dihydrothymine in bodily fluids [10]. Thereafter, several other patients have been reported with DHP deficiency based on elevated levels of dihydropyrimidines, thymine and uracil in their blood, urine and cerebrospinal fluid. In 1997 Assman et al. reported a Turkish boy from a consanguineous marriage with significant elevations of dihydropyrimidins, thymine and uracil in urine, suggesting a pyrimidine degradation disorder. He presented intractable diarrhea as a result of congenital microvillus atrophy. He also developed severe cholestasis which led to liver cirrhosis. His liver biopsy demonstrated an enzymatic defect of 5,6-dihydropyrimidine amidohydrolase [14]. Elevated levels of urinary dihydropyrimidines, cryptogenic cirrhosis and increased levels of liver enzymes were observed in our patient.

Genetically, compound heterozygous or homozygous mutations in the *DPYS* gene can cause DHP deficiency. According to the Human Gene Mutation Database (HGMD), there are 18 missense/nonsense mutations (2 nonsense), 4 small deletions and 1 small insertion have been reported in *DPYS* gene until now. In this study, we report another pathogenic stop gain variant which alters a lysine amino acid to premature stop codon at codon 501 leading to truncation of DHP protein. Our patient is homozygous for this mutation (Exon 9, c.1501 A > T, p.K501X) which cause DHP deficiency.

Since *DPYS* is only expressed in liver and kidney, it is difficult to study the effects of mutations in this gene. Fortunately, we had patient’s liver tissue specimen, which enabled us to evaluate the mRNA expression level of *DPYS* gene in our patient’s affected hepatic tissue. The expression level of DPYS in patients tissue showed a significant decrease compared to a healthy liver tissue which proved the disease causing status of c.1501 A > T (p.Lys501Ter) mutation in our patient.

In summary, in the current study a novel stop gain mutation in *DPYS* gene was identified in a patient affected with DHP deficiency in southern Iran. This private mutation has not been previously reported. In addition the effects of this mutation on exon 9 of *DPYS* gene was confirmed using bioinformatics tools, Sanger sequencing and Real Time PCR analysis of the patient’s liver biopsy specimen. Our findings proved the pathogenicity of this novel stop gain mutation in *DPYS* gene. Although there is not any specific treatment for patients with DHP deficiency, diagnosis of this disorder can help providing more accurate genetic counseling for high risk individuals and aids prevention of this disorder in families with affected individuals.

## Data Availability

All relevant data are included in the manuscript. The sequencing datasets generated or analyzed during the current study are not publicly available because individual privacy could be compromised. The datasets are available from the corresponding author upon reasonable request. The dataset corresponding to the DPYS gene can be found in NCBI under the accession number ENST00000351513. The dataset corresponding to the DPYS mRNA sequence can be found in NCBI under the accession number NM_001385. The following links have been used to analyze the patient’s data: Human reference genome (GRCH37/hg19) (https://www.ncbi.nlm.nih.gov/assembly/GCF_000001405.13/), Ensemble Variant Effect Predictor (VEP) (https://asia.ensembl.org/info/docs/tools/vep/index.html), ClinVar (https://www.ncbi.nlm.nih.gov/clinvar/), OMIM (https://www.ncbi.nlm.nih.gov/omim), GWAS (http://www.ebi.ac.uk/gwas/), HGMD (http://www.hgmd.cf.ac.uk/ac/index.php), SwissVar (https://swissvar.expasy.org/), 1000 Genome phase 3 (https://www.internationalgenome.org/category/phase-3/), ExAC (http://exac.broadinstitute.org/), EVS (https://evs.gs.washington.edu/EVS/), dbSNP (http://www.ncbi.nlm.nih.gov/SNP/), Polyphen 2 (http://genetics.bwh.harvard.edu/pph2/), SIFT (https://sift.bii.a-star.edu.sg/), Mutation Taster 2 (http://www.mutationtaster.org/), Mutation Assessor (http://mutationassessor.org/r3/).

## Abbreviations

DHP: Dihidropyrimidinase
DPD: Dihydropyrimidine Dehydrogenase
ALT: Alanine transaminase
AST: Aspartate transaminase
Alk-P: Alkaline phosphatase
PT: Prothrombin time
HPLC: High Performance Liquid Chromatography
WES: Whole-Exome Sequencing
NGS: Next generation sequencing
ANA: Anti-nuclear antibody
ASMA: Anti-smooth muscle antibody
